# Novel Insights into Microbial Behavior Gleaned Using Microfluidics

**DOI:** 10.1264/jsme2.ME22089

**Published:** 2023-03-23

**Authors:** Kohei Takahashi, Xiaojie Li, Tatsuki Kunoh, Ryo Nagasawa, Norio Takeshita, Andrew S. Utada

**Affiliations:** 1 Graduate School of Life and Environmental Sciences, University of Tsukuba, Tsukuba, Ibaraki, Japan; 2 Faculty of Life and Environmental Sciences, University of Tsukuba, Tsukuba, Ibaraki, Japan; 3 Microbiology Research Center for Sustainability, University of Tsukuba, Tsukuba, Ibaraki, Japan

**Keywords:** microfluidics, biofilm, *P. aeruginosa*, *S. mutans*, *L. cholodnii*, filamentous fungi

## Abstract

Microorganisms develop into communities in nearly every environmental niche, which is typically replete with micrometer-scale gaps and features. In each of these habitats, microorganisms adapt to and are affected by their physical environment. Conventional culture methods use glass bottom dishes or millimeter-scale flow cells, which poorly mimic the complexity of natural micrometer-scale environments; therefore, the limitations associated with the creation of microbe-scale environments with granularity hinder the ability to examine their ecological behavior. Microfluidics is a tool that is increasingly being used to study microorganisms because it enables the manipulation of micrometer-scale flows while simultaneously facilitating real-time and live-cell imaging. In this review, we discuss several insights into the behavior of bacteria and fungi that were gained through the adoption of microfluidics to control complex micrometer-scale environments. We also discuss the potential of the increased adoption of this tool.

An estimated 10^30^ microorganisms live on Earth and inhabit nearly every conceivable niche (Raynaud and Nunan, 2014; Flemming and Wuertz, 2019), and the number of microorganisms in soil and aquatic environments was recently estimated to be ~5×10^29^. In soil and aquatic environments, microorganisms primarily inhabit agglomerated inorganic and sticky organic matter (Hopper, 2009; Louca *et al.*, 2019), which may take the form of solid/semi-solid flocs in the water column; tortuous paths with numerous dead-end pockets that retain water in hydrated soils; or the pockets between the tooth surface and gingiva in ‘higher’ organisms. In most of these environments, microorganisms inhabit spaces with a geometric texture ranging between ~1 and 100‍ ‍μm, and are physically determined by the particles in and features of these structures (Kim and Or, 2016).

The dominant lifestyle for prokaryotic microorganisms is a surface-attached biofilm (Flemming and Wuertz, 2019), which is a dense community of cells that adhere to a submerged surface. The biofilm lifestyle is advantageous for inhabitants because it facilitates the synchronization of gene expression through proximity-based cell-cell communication, enables nutrient sharing, and provides protection from a variable environment as well as physical and chemical attacks (Donlan and Costerton, 2002; Fux *et al.*, 2005; Nguyen *et al.*, 2011). In addition to these benefits, subpopulations in biofilms spontaneously develop heterogeneity in the form of distinct metabolic pathways and stress responses (Stewart and Franklin, 2008; Nguyen *et al.*, 2011) that may lead to a significant increase in antibiotic resistance. Biofilm development starts with the ‘irreversible’ attachment of a single bacterium to an environmental substrate (van Loosdrecht *et al.*, 1990; Persat *et al.*, 2015; Secchi *et al.*, 2020). In environments that support growth, cell division leads to the formation of biofilms (Mah *et al.*, 2003; Stewart and Franklin, 2008), which utilize self-secreted extracellular polymeric substances (EPS) composed of polysaccharides, proteins, and extracellular DNA (eDNA) to ensure that the community remains adhered to a surface (Das *et al.*, 2010; Kawarai *et al.*, 2016; Jung *et al.*, 2017; Nagasawa *et al.*, 2020a). This paradigm of biofilm formation is generally applicable in any colonizable environment (Flemming and Wuertz, 2019); however, the specific mechanism underlying biofilm formation strongly depends on the physical characteristics of the ecological niches occupied by bacteria.

Although eukaryotic microorganisms, primarily yeasts and fungi, may also form biofilms, this phenomenon has primarily been examined in the clinical context with yeasts (Miranda *et al.*, 2022). Filamentous fungi account for the vast majority of eukaryotic microorganisms inhabiting the subsurface (Osono *et al.*, 2003; Fierer, 2017), and it currently remains unclear whether these organisms follow a similar biofilm-forming program. Filamentous fungi grow into mycelial networks of filamentous hyphae that burrow directly through the soil (Abeysinghe *et al.*, 2020; Fukuda *et al.*, 2021). Fungal mycelial networks are crucially important for soil health and, consequently, for ecological health (Nazir *et al.*, 2010). These organisms cohabit many of the same environments as bacteria and, thus, demonstrate both cooperative and antagonistic behaviors mediated by secreted chemical signals for communication (Abeysinghe *et al.*, 2020). Interactions between microorganisms in such a crucial environment as soil may be examined using methods that accurately reconstruct this environment in a laboratory setting.

A major limitation to the study of microorganisms is the difficulty associated with recreating the physical environments that they inhabit. Since confinement by local crowding from other organisms or by environmental structures directly affects behavior (Boedicker *et al.*, 2009; Hochbaum and Aizenberg, 2010; Drescher *et al.*, 2013), the ability to impose and control geometry is important. Microbial behavior is typically imaged and analyzed in medium-filled plastic wells or Petri dishes. Although culture methods are simple and relatively inexpensive, there are a number of significant disadvantages that impact their usefulness. They quickly experience limitations in nutrients due to the closed system and have uncontrolled currents due to thermal convection, both of which may affect the behavior of microorganisms (Dijkstra *et al.*, 2011). Flow cells, which are millimeter-scale flow channels, overcome the issues of variable nutrient conditions and have been employed for cell‍ ‍tracking and biofilm development (Foster and Kolenbrander, 2004; Zhao *et al.*, 2013; Utada *et al.*, 2014). Although various disposable flow cells are currently available and easy to use, they fail to reproduce the micrometer-scale gaps and features utilized by microorganisms in the environment that may be formed using microfluidics.

Microfluidics involves the manipulation and control of micro-scale fluid flows (Xia and Whitesides, 1998; Bocquet and Charlaix, 2010), and has been increasingly used over the past 15 years to investigate environmental microorganisms. Microfluidic technology has enabled new methods of ana­lysis and increased the throughput of iterative testing in chemistry, physics, biology, and biotechnology (Utada *et al.*, 2005; Macosko *et al.*, 2015; Lan *et al.*, 2017). Microfluidic devices are commonly fabricated using a method called soft lithography, which employs photolithography to generate a mold in-relief that is then used to emboss a soft material, typically the cross-linkable polymer polydimethylsiloxane (PDMS) (Zhao *et al.*, 1997). PDMS has the benefits of being: 1) transparent, facilitating imaging; 2) highly permeable to oxygen, facilitating the growth of aerobic organisms; 3) relatively inexpensive; and 4) easily bonded to glass, which enables the capping of the embossed pattern to form closed microchannels. Although the facilities needed to fabricate these devices may be uncommon in environmental microbiology laboratories, the majority of engineering departments have the necessary facilities. In addition, disposable microfluidic chips are now commercially available, further simplifying the adoption of this method.

The precise control afforded by microfluidics has facilitated advances in research on microorganisms (Balaban *et al.*, 2004; Liu *et al.*, 2015). Microfluidics has enabled the tracking of individual microorganisms within communities and has, thus, allowed microbiologists to address fundamental questions about the mechanisms by which microorganisms form communities as well as their evolution over time within these environments. The use of microfluidic technology also permits observations of microbial community development in environments that more closely mimic natural environments (Stanley *et al.*, 2016; Aleklett *et al.*, 2018). These studies demonstrated the in-roads that microfluidics have made in the study of microorganisms and the adoption of microfluidic techniques by microbiologists.

We herein review our recent efforts utilizing microfluidics to investigate a wide range of environmental microorganisms. We discuss methods to disrupt biofilms of the opportunistic pathogen *Pseudomonas aeruginosa* PAO1 from channels as a model for biofilm clearance from sensitive or delicate regions for which toxic chemicals cannot be‍ ‍used. We examine the use of microfluidic confinement to‍ ‍facilitate the ana­lysis of single-cell behavior in dense 3D‍ ‍biofilms of the dental caries-promoting bacterium *Streptococcus mutans*. We demonstrate the versatility of this device by applying it to investigations on the dynamics of filamentation by *Leptothrix cholodnii*, which is responsible for problematic bulking in wastewater treatment plants. We also show the utility of microfluidic confinement to investigate the dynamics of hyphal growth in filamentous fungi.

## Section 1: Microfluidics enables the parallel quantification of mature biofilm removal

Biofilm formation in undesirable locations is often a nuisance and may lead to serious and costly problems. Biofilm formation on indwelling devices and implants has been shown to cause chronic infections (Subhadra *et al.*, 2018). It may also foul surfaces, leading to corrosion, and clog the pores of the reverse osmosis membranes of potable water systems (Whittaker *et al.*, 1984). Strong oxidizing agents are used to disrupt biofilms, but are not deployable on delicate substrates, such as living tissue, and may also degrade the underlying substrate over time.

**Fig. 1.**
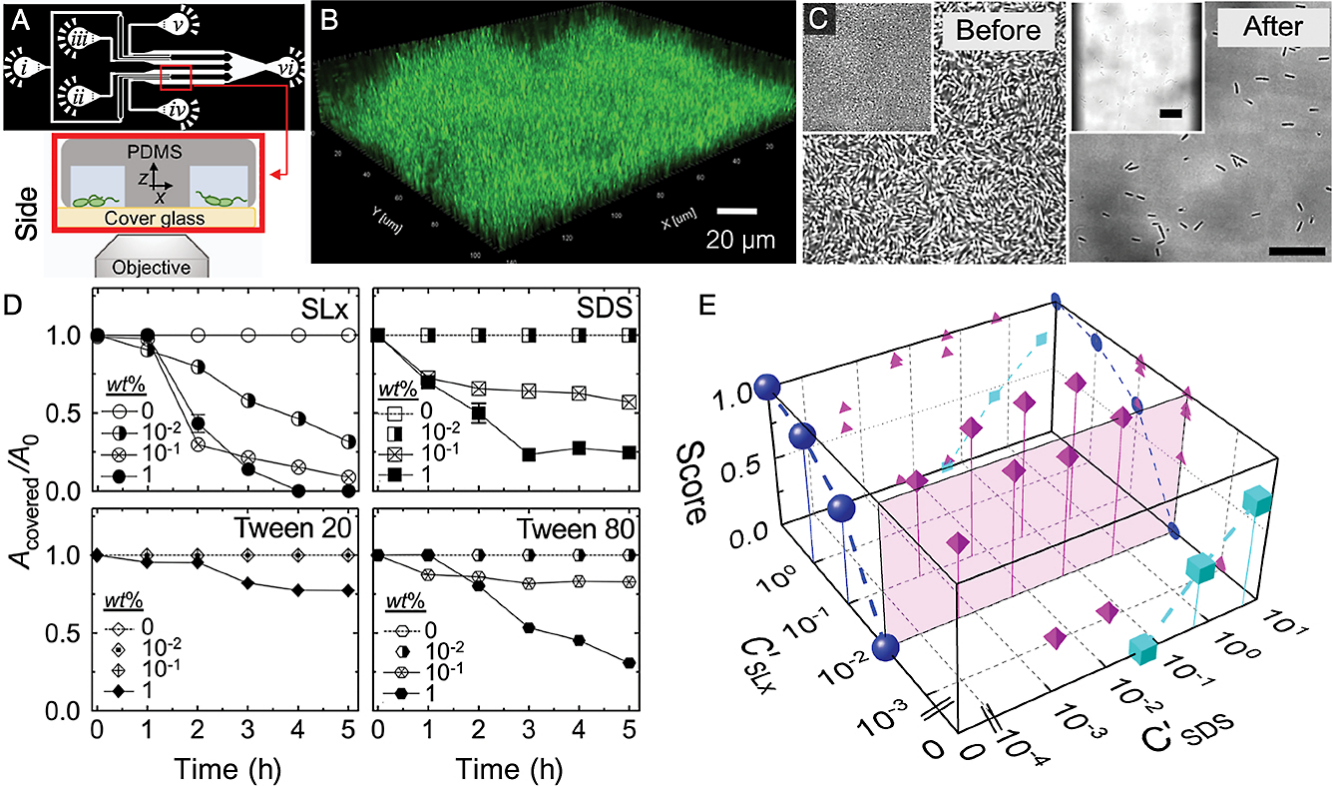
Quantitative assessment of the effect of surfactants on biofilm disruption in parallel growth channels. (A, upper) Microfluidic channel design. Inlet (*i*) is used to inoculate each channel with the same culture, inlets (*ii, iii, iv, and v*) are used to infuse different solutions, and (*vi*) is the outlet. (A, lower) Cross-sectional schematic of two channels. (B) Confocal image of a typical mature PAO1 biofilm taken at 12 h. (C) Bright-field images of channels before and after a treatment with 0.1 wt% sophorolipid solution. (insets) The whole channel is shown, which has dimensions of 133×133‍ ‍μm^2^. Scale bars=10‍ ‍μm. (D) Surface area covered by the biofilm (*A*_covered_), normalized by the total area (*A*_0_) of the field of view, measured as a function of the surfactant treatment time. The symbols represent different surfactant concentrations. (E) Biofilm disruption score (*A*_covered_/*A*_0_) at *t*=5‍ ‍h as a function of the normalized concentrations of SL and SDS, *C*’_SL_ and *C*’_SDS_, respectively. The score ranges from 0–1, with 0 representing zero removal and 1 indicating complete removal. SL and SDS concentrations were normalized by their respective critical micelle concentrations. Adapted from Ref. (Nguyen *et al.*, 2020)

Biofilm disruption has traditionally been measured using plate-based assays (Guilhen *et al.*, 2017); however, these methods provide one-dimensional information on color, optical density, and fluorescence and limited insights into biofilm dynamics. Using multiple closely-spaced microfluidic channels, we examined the effects of the glycolipid biosurfactant sophorolipid (SL) on biofilms of *P. aeruginosa* PAO1, which we used as a model for a pathogenic biofilm-forming bacterium (Fig. 1A) (Nguyen *et al.*, 2020). This approach allowed for the testing of biosurfactants, which are surface-active molecules secreted by various bacteria and fungi that are less toxic than typical chemical surfactants and may have novel characteristics due to their origin (Baccile *et al.*, 2017).

We deliberately designed our device to facilitate the simultaneous inoculation of all channels as well as the easy repositioning of the microscope field-of-view for rapid image acquisition in all channels. The continuous flow of media enabled the development of biofilms in each channel, which were then tested with different surfactants (Fig. 1B and C). Time-lapse imaging enabled us to quantitatively track the evolution of biofilms in the presence of different surfactants, which also permitted measurements of dose dependency (Fig. 1D).

Importantly, our devices allowed us to simultaneously test many conditions, which decreased the burden on microscope usage. Furthermore, we detected synergy between SL and the anionic surfactant sodium dodecyl sulfate (SDS); the combination of these two surfactants enhanced the effectiveness of each over that when they were used separately. We generated a phase diagram that combined the concentrations of SL and SDS in the growth medium, with a biofilm disruption score ranging between 0 and 1, where 0 indicates no disruption and 1 equals complete removal (Fig. 1E). This phase diagram showed the clear benefit of combining the two surfactants (see magenta icons). Moreover, without the ability to parallelize data acquisition, this type of test may have been difficult. In principle, different biofilm-forming bacteria may be easily substituted, and additional channels may also be easily plumbed in to further increase throughput.

## Section 2: 2D microfluidics enable imaging of eDNA production in *S. mutans*

**Fig. 2.**
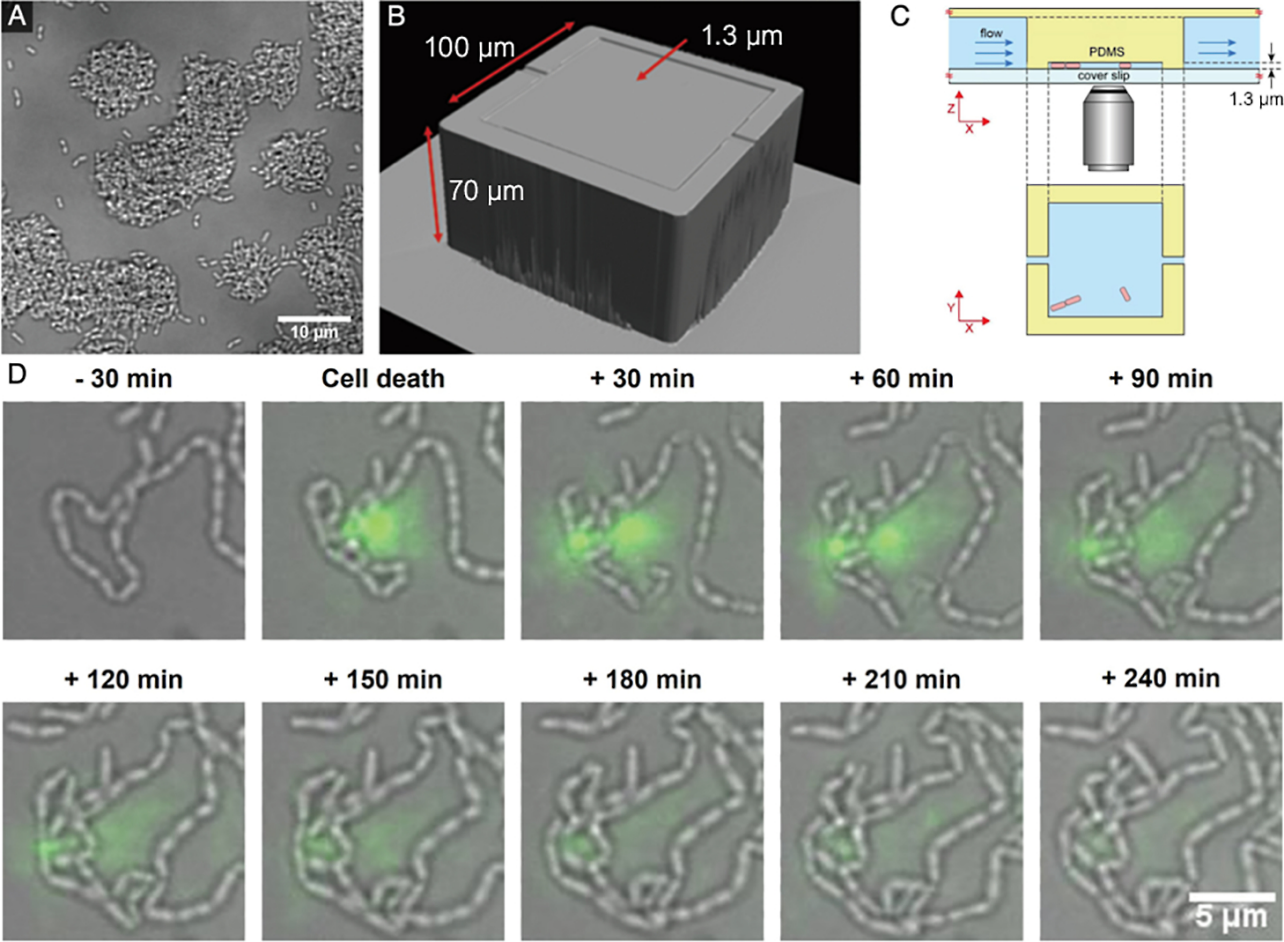
2D confinement of *S. mutans* colonies. (A) Bright-field image of dense 3D microcolonies of *S. mutans* growing under unconfined conditions. (B) Isometric view of the high-aspect ratio microfluidic chamber. The glass cover slip that caps the device is omitted for clarity. Cells are cultured and imaged in the 1.3-μm gap. (C) Schematic showing the side and top views of the chamber within the larger flow channel (not to scale). The square chamber is the top view of the chamber shown in (B). (D) Bright-field sequence with overlaid fluorescence images showing *S. mutans* UA159 growing in a 2D chamber with the release of eDNA (green) labeled with SYTOX Green. Adapted from Refs. (Kunoh *et al.*, 2020; Nagasawa *et al.*, 2020b)

In addition to parallelization, channels with different heights, ranging between ~100 and <1‍ ‍μm, may be fabricated using similar methods (Rotem *et al.*, 2012). Since biofilms are dense communities with 3D structures, it may be challenging to resolve individual cells inside a biofilm (Fig. 2A). We demonstrated how microfluidics may be used to confine bacteria to thin layers in high-aspect ratio gaps, which we call 2D microchambers (Fig. 2B and C). This confinement facilitates the tracking of cell fate with high spatio-temporal resolution imaging by preventing cells from piling on top of each other. This tracking of cell fate is important in investigations on phenotypic heterogeneity, such as the division of labor within a biofilm, the appearance of persister cells, and elucidating how and where cell death occurs (Turnbull *et al.*, 2016; Armbruster *et al.*, 2019; Personnic *et al.*, 2021).

eDNA is an important component of the EPS matrix in *S. mutans* biofilms and is necessary for initial cell attachment; however, the mechanisms by which cells release eDNA from within a biofilm are unclear. By culturing *S. mutans* in our 2D microchambers, we imaged the release of eDNA into the extracellular space, which we were correlated with cell death (Nagasawa *et al.*, 2020b) (Fig. 2D). We noted a variation in the release of eDNA from cells labeled as dead, which suggested a morphological variation in the manner of bacterial cell death. This warrants further study.

Based on our studies, it appears that eDNA is also an important common factor. We demonstrated that 2D confinement offers the opportunity to test conditions that may otherwise be impossible to recreate in a controlled manner, which may reveal cell affinity for particular environmental geometries (Nagasawa *et al.*, 2020b).

## Section 3: 2D confinement reveals dynamics of filament formation in *L. cholodnii*

Physical confinement is a useful method for enforcing limitations on vertical growth away from the imaging plane, which is important when imaging biofilms, but is also useful for imaging whole-colony dynamics in the case of filamentous bacteria. Filamentous bacteria, which form long cell chains, rapidly grow out of the focal plane, hindering the imaging of filament dynamics. These bacteria form macroscopic 3D biofilms, called biomats, and cause bulking, which may significantly reduce wastewater plant efficiency (Krumbein *et al.*, 2003; Chan *et al.*, 2016). The development of methods to control their growth requires a detailed understanding of the dynamics of filament formation.

**Fig. 3.**
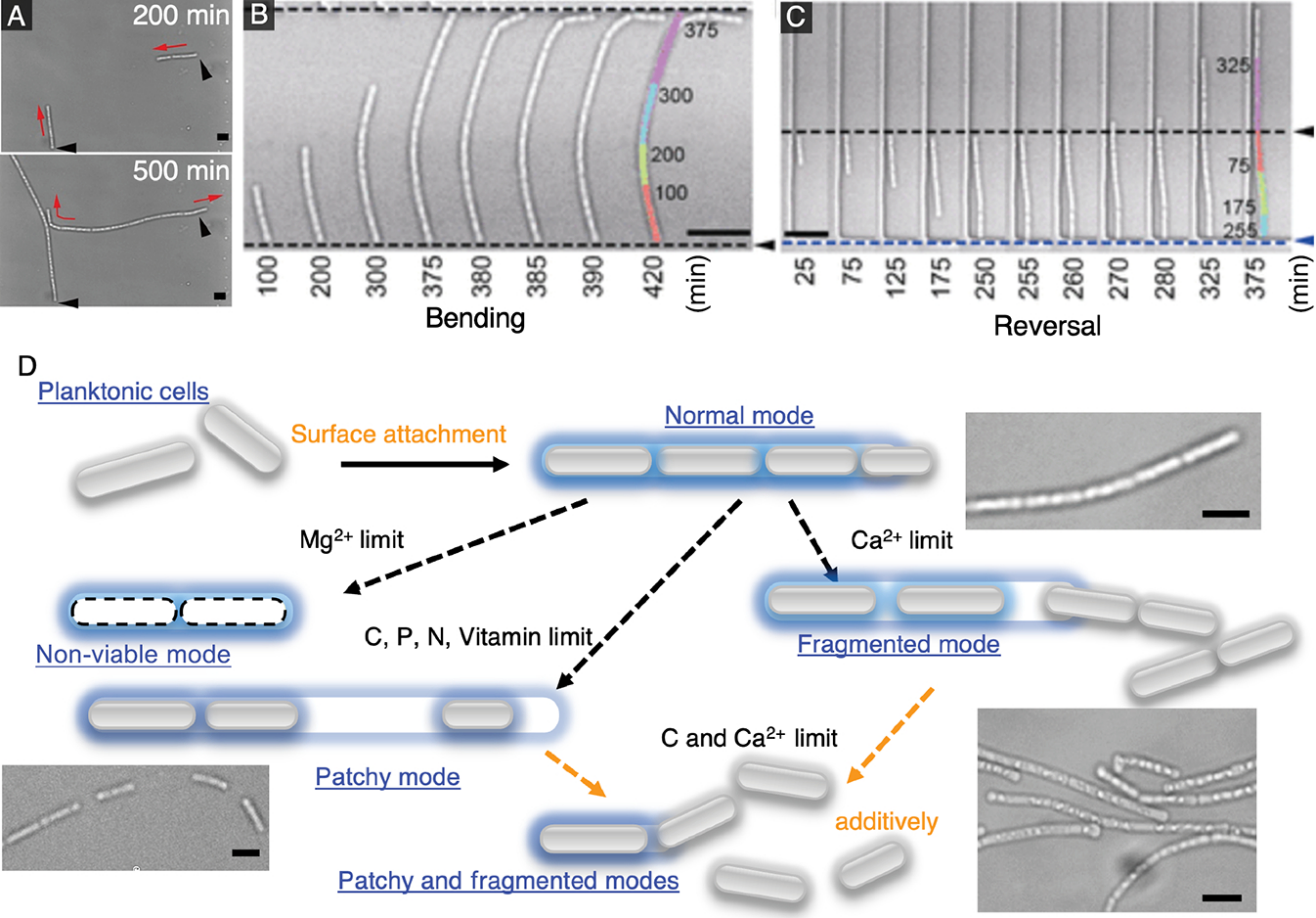
*Leptothrix* filament dynamics imaged in 2D chambers. (A) Bright-field images showing (upper) unilateral and (lower) bilateral elongation in the chamber. (B and C) Time sequence showing filament bending and filament reversal. The colored segments show the filament shape at the indicated times overlaid on the filament in the last frame. (D) Schematic and images showing the four modes of filament development under nutrient deficient conditions. Scale bars=5‍ ‍μm. Adapted from Refs. (Kunoh *et al.*, 2020, 2021)

Using a similar 2D device to that described earlier, we cultured the filament-forming bacterium *L. cholodnii* in 2D microchambers to analyze their filamentation dynamics (Kunoh *et al.*, 2020). *Leptothrix* form filaments encased in a sheath composed of nanofiber-like appendages called nanofibrils (Van Veen *et al.*, 1978). By tracking individual cells combined with the *in situ* labeling of nanofibrils, we showed that nanofibrils were not only essential for irreversible surface attachment, but also affected the direction of filamentation. Elongation was unilateral when nanofibrils capped the cell pole, and was bilateral when they surrounded the cell waist (Fig. 3A).

Confined conditions also allowed us to analyze how filaments reacted upon collision with obstacles. We found that elongating filaments either bent or reversed direction when they collided with obstacles (Fig. 3B and C). We estimated the forces on filaments from bending dynamics and noted a strong dependence on the angle of collision between filaments and the obstacle. A filament was more likely to bend with a shallow angle of collision, but reversed directions with a larger angle. In addition, we observed the appearance of intercellular gaps within elongating cell chains as well as cell escape from the elongating pole(s). These characteristics implied that local environmental conditions, such as the‍ ‍availability of dissolved nutrients or minerals, affect *Leptothrix* behavior (Chan *et al.*, 2016; Kunoh *et al.*, 2020).

After clarifying single-filament dynamics, we investigated methods to control and modify the sheath, which may lead to practical methods for the management of bulking. We used microfluidics to exchange growth media quickly and easily in microfluidic devices, thereby permitting measurements of the effects of the abrupt limitation of different micronutrients on filament behavior (Kunoh *et al.*, 2021). The findings obtained revealed that filament development under nutrient-deficient conditions may be classified into four general modes: (i) “normal”, where the cell chain elongates without the appearance of intercellular gaps; (ii) “patchy”, where large intercellular gaps appear within cell chains; (iii) “non-viable”, where cell autolysis occurs before cell division may proceed; and (iv) “fragmented”, where cell chains split into smaller chains (Fig. 3D). Among all of the micronutrients tested, we found that the limitation of carbon or calcium exerted the strongest effects on filaments. The removal of carbon resulted in partially filled filaments with a low cell density, while the removal of calcium led to filaments that fragmented. Moreover, the removal of both nutrients induced the simultaneous onset of “patchy” and “fragmented” modes, which essentially arrested filament formation.

As proof of concept, our device allows for the characterization of microbial behavior, which will provide insights that will contribute to the control of their growth. By using this method, rather than the addition of toxic chemicals to kill organisms that cause bulking, we demonstrated that the removal of key nutrients may weaken filaments, which may create conditions for the natural resolution of this issue.

## Section 4: Hyphal growth in pore-like channels

As observed with *Leptothrix*, microfluidic confinement may facilitate the tracking and ana­lysis of organisms that escape the imaging plane. We described the use of microfluidics to investigate cell polarity and growth in filamentous fungi, which are fundamental processes for all cellular functions (Howard *et al.*, 2011; Zhao *et al.*, 2018). Since the core machinery controlling cell polarity appears to be relatively conserved across eukaryotes (Asnacios and Hamant, 2012; Campanale *et al.*, 2017), filamentous fungi are a tractable model system for investigations on these processes.

**Fig. 4.**
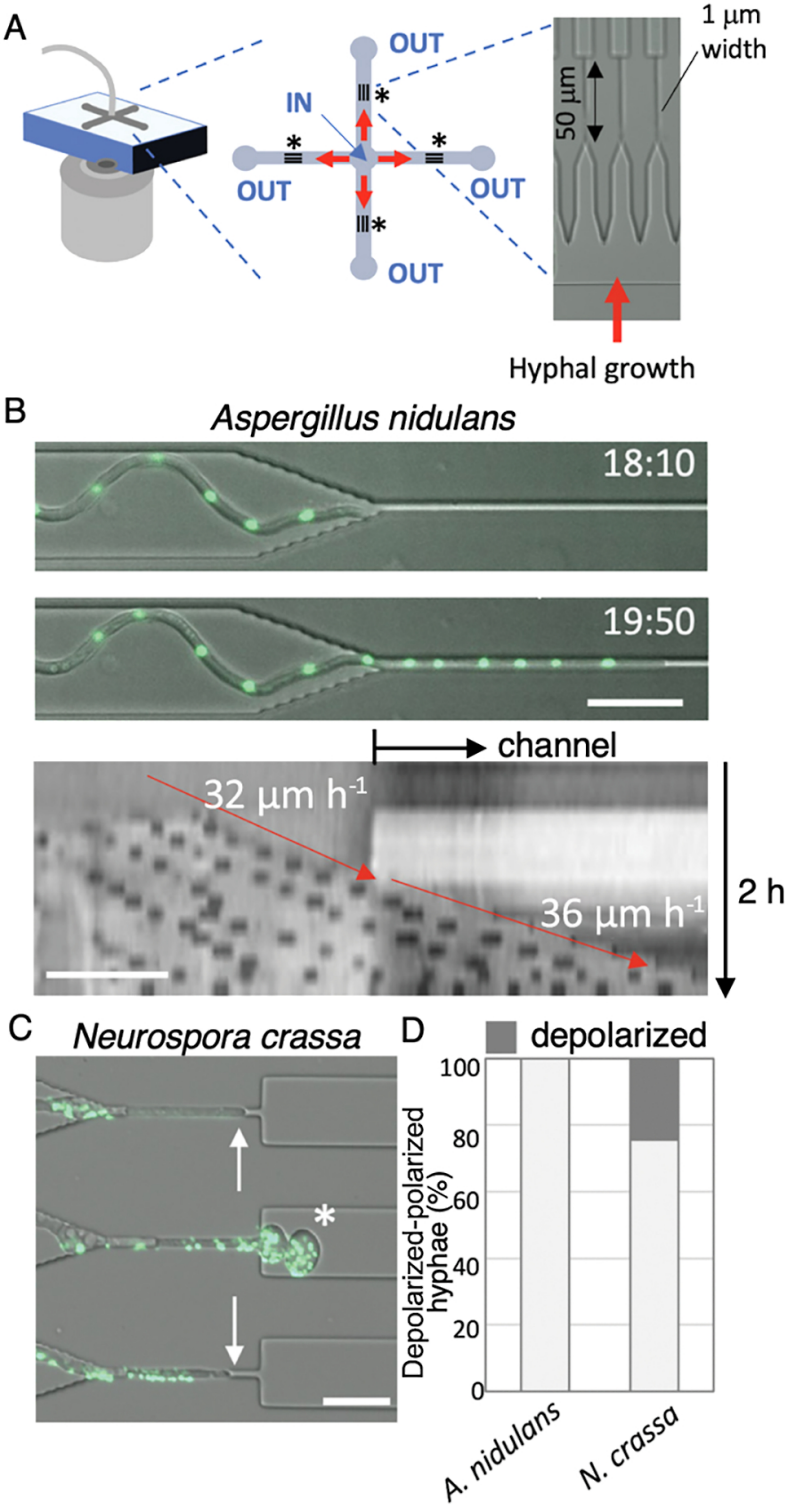
Imaging hyphal growth through pores. (A) Schematic of the microfluidic device used. The central input is used to inoculate and infuse the medium, while the four ports at the ends of the cross (labeled “out”) are used as outlets. Twenty 1×50‍ ‍μm microchannels were designed between the inlet and outlet for each branch. (B, upper) Time sequence showing the growth of a hypha of *Aspergillus nidulans* (nuclei labeled with GFP). The elapsed time is given in h:min. (B, lower) A kymograph along the growth axis before and inside the channel. Hyphal elongation rates prior to and after entry into the channel are shown as black slopes (and red arrows). (C) Time sequence showing the stoppage of growth of the hyphae of *Neurospora crassa* (nuclei labeled with GFP). The location at which growth stops is indicated by the arrows. Depolarized hyphae exiting from the channels are indicated with asterisks. Scale bars=20‍ ‍μm. (D) Rate of polarized and depolarized hyphae that passed through the channels for *A. nidulans* and *N. crassa* (*n*=50 for each). Adapted from Refs. (Fukuda *et al.*, 2021)

Filamentous fungal cells develop into highly polarized tubular structures that elongate through the continuous supply of membrane lipids and the *de novo* synthesis of cell wall material at the extending tip (Riquelme *et al.*, 2018); however, the mechanisms by which growth speed and cell polarity cooperatively control cell shape remain unclear. We speculated that insights into this relationship may be obtained by forcing fungal hyphae to grow through gaps narrower than the natural diameter of the hyphal tip (Fukuda *et al.*, 2021). Therefore, we designed pore-like channels of a sufficient length that combined both vertical and lateral microfluidic confinement (Fig. 4A).

We measured hyphal growth speed through these channels using the slower and faster growing fungi, *Aspergillus nidulans* and *Neurospora crassa*, respectively. We found that *A. nidulans*, the typical hyphal width of which was ~3‍ ‍μm before entering the narrow channel, appeared to traverse the pore and continued elongating (Fig. 4B, upper). A kymograph of the growth axis of a representative hypha showed similar growth rates before and after entering the narrow pore; the speed of the elongating tip is indicated by red arrows (Fig. 4B, lower). In contrast, *N. crassa*, the hyphal width of which was ~4‍ ‍μm, exhibited a different behavior. In some cases, hyphae stopped elongating in the pore or paused frequently before traversing it. In other cases, hyphae fully traversed the channel, but often showed a loss of tip polarization, which led to the localized “balling-up” of the tip (Fig. 4C). These depolarized hyphae eventually stopped growing. The findings of these experiments revealed that fast-growing hyphae (*N. crassa*) often lost cell polarity after emerging from the narrow channels, whereas slow-growing hyphae (*A. nidulans*) retained their polarity, as shown in Fig. 4D. We examined seven different filamentous fungi using the same approach and obtained similar findings. Our experiments generally showed that fast-growing hyphae more frequently lost cell polarity after emerging from the narrow channels, whereas slow-growing hyphae retained their polarity (Fukuda *et al.*, 2021), highlighting a trade-off between plasticity and velocity in hyphal growth.

## Conclusion

In this review, we discussed the insights obtained into the behavior of environmental microorganisms utilizing geometrical confinement in microfluidic channels. The studies outlined herein have not only contributed to a more detailed understanding of biofilm formation and promising biofilm management strategies, but also indicate the potential of utilizing microfluidics for fundamental cell biology. The additional control of the physical environment over that achieved in microtiter plates and flow cells provides further means to probe and analyze microbial behavior. Our experiments, which take advantage of the ability to control spatial dimension, offer insights into microbial behavior in a manner that was not possible in previous research. In the areas of life sciences and biomedical engineering, the flexibility to design and control a local environment has been utilized to create increasingly sophisticated organ-on-a-chip devices (Bhatia and Ingber, 2014), which are enabling the study of organogenesis in controlled environments. A similar approach is being adopted in microbiology with soil-on-a-chip devices (Stanley *et al.*, 2016) that attempt to mimic the environment or manage interkingdom interactions in a controlled manner, which will aid in the study of microbial ecology. We anticipate that the techniques and infrastructure needed to fabricate and operate microfluidic devices will become more widely available, increasingly important, and make fundamental contributions to microbial ecology.

## Citation

Takahashi, K., Li, X., Kunoh, T., Nagasawa, R., Takeshita, N., and Utada, A. S.. (2023) Novel Insights into Microbial Behavior Gleaned Using Microfluidics. *Microbes Environ ***38**: ME22089.

https://doi.org/10.1264/jsme2.ME22089
